# Multi-task reinforcement learning and explainable AI-Driven platform for personalized planning and clinical decision support in orthodontic-orthognathic treatment

**DOI:** 10.1038/s41598-025-09236-z

**Published:** 2025-07-08

**Authors:** Zhiyuan Li, Liwei Wang

**Affiliations:** Department of Stomatology, General Hospital of Northern Theater Command, Shenyang, 110002 Liaoning China

**Keywords:** Multi-task reinforcement learning, Explainable artificial intelligence, Orthodontic-orthognathic treatment, Clinical decision support, Personalized medicine, Biomechanical simulation, Information technology, Dental diseases, Oral diseases

## Abstract

This study presents a novel clinical decision support platform for orthodontic-orthognathic treatment that integrates multi-task reinforcement learning with explainable artificial intelligence. The platform addresses the challenges of personalized treatment planning in complex dentofacial deformities by formulating treatment as a sequential decision-making process optimizing multiple clinical objectives simultaneously. We developed a comprehensive framework comprising: (1) a multi-task reinforcement learning core with specialized state-action representations for craniofacial structures; (2) complementary explainable AI components that render complex model decisions interpretable within clinical contexts; and (3) an interactive interface facilitating collaborative human-AI decision-making. Experimental validation with 347 retrospectively analyzed cases demonstrated significant improvements in treatment plan quality (19.9%), decision efficiency (73.9% time reduction), and prediction accuracy (92.7%) compared to conventional methods. Clinical evaluation by multidisciplinary specialists confirmed the system’s practical utility, with particularly strong performance in complex cases featuring multiple dentofacial abnormalities. The proposed platform represents a significant advancement toward evidence-driven, personalized treatment planning in orthodontic-orthognathic therapy while maintaining clinical interpretability and expert oversight.

## Introduction

Orthodontic-orthognathic combined treatment represents an interdisciplinary approach for managing severe dentofacial deformities that cannot be adequately addressed through orthodontic treatment alone^1^. This complex therapeutic modality integrates orthodontic techniques with orthognathic surgical procedures to achieve optimal functional and aesthetic outcomes in patients with significant skeletal discrepancies^2^. Despite its effectiveness, the planning and execution of orthodontic-orthognathic combined treatment presents numerous clinical challenges that significantly impact treatment success and patient satisfaction.

The complexity of personalized treatment planning in orthodontic-orthognathic cases stems from the multifactorial nature of dentofacial deformities, encompassing skeletal, dental, and soft tissue components that vary substantially among individuals^3^. Clinicians must integrate and analyze large volumes of heterogeneous data, including cephalometric measurements, three-dimensional facial scans, dental models, and patient-specific functional and aesthetic requirements^4^. Traditional planning methods rely heavily on clinician experience and subjective judgment, which can lead to inconsistent treatment decisions and suboptimal outcomes in complex cases^5^.

Furthermore, the decision-making process in orthodontic-orthognathic treatment involves navigating numerous interdependent clinical parameters and predicting post-treatment outcomes across multiple time horizons^6^. These decisions carry significant implications for treatment duration, potential complications, and ultimately, patient quality of life^7^. Conventional approaches often fail to fully account for the dynamic nature of treatment response and the intricate relationships between different treatment variables, resulting in treatment plans that may require significant adjustments during implementation.

Recent advances in artificial intelligence, particularly multi-task reinforcement learning (MTRL), offer promising solutions to these challenges by enabling simultaneous optimization of multiple treatment objectives^8^. MTRL algorithms can learn optimal treatment strategies from clinical data while balancing competing goals such as treatment efficiency, stability, and aesthetic outcomes. Complementing this approach, explainable artificial intelligence (XAI) methods address the “black box” problem inherent in many AI systems by providing transparent reasoning behind AI-generated recommendations^9^. These interpretable models are essential for building clinician trust and facilitating integration into clinical workflows.

Our proposed clinical decision support platform leverages the synergistic combination of MTRL and XAI to overcome existing limitations in orthodontic-orthognathic treatment planning^10^. This innovative system aims to enhance clinical decision-making through personalized treatment simulations, outcome predictions, and explainable recommendations that account for individual patient characteristics. By augmenting clinician expertise with AI-driven insights, the platform seeks to optimize treatment efficiency, improve predictability of outcomes, and ultimately enhance patient satisfaction in complex orthodontic-orthognathic cases.

## Literature review and theoretical foundation

### Current status analysis of orthodontic-orthognathic combined treatment

Orthodontic-orthognathic combined treatment has evolved significantly since its formal recognition as a specialized interdisciplinary approach in the mid-20th century^11^. Traditional treatment planning methodologies were primarily based on two-dimensional cephalometric analyses, which provided limited information about complex three-dimensional dentofacial deformities^12^. These conventional approaches relied heavily on standardized reference values and manual prediction tracing, often failing to account for individual variations in skeletal patterns, soft tissue dynamics, and functional needs^13^.

The integration of digital technologies into orthodontic-orthognathic treatment workflows marked a significant advancement in the field, with cone-beam computed tomography (CBCT) enabling comprehensive three-dimensional assessment of craniofacial structures^14^. Computer-aided surgical simulation (CASS) further revolutionized treatment planning by allowing virtual surgical manipulations and outcome predictions, reducing reliance on subjective clinical judgment^15^. Despite these technological innovations, substantial challenges persist in translating digital information into optimized treatment strategies that balance functional improvement, aesthetic outcomes, and long-term stability.

Current clinical decision-making in orthodontic-orthognathic cases remains predominantly experience-based rather than evidence-driven, with considerable variation in treatment approaches among clinicians facing similar clinical scenarios^16^. This variability stems from the absence of standardized decision protocols that can accommodate the heterogeneity of dentofacial deformities and patient-specific factors including age, growth patterns, and psychosocial considerations^17^. Furthermore, the complexity of predicting surgical outcomes is compounded by the dynamic interplay between orthodontic tooth movement, skeletal repositioning, and subsequent soft tissue adaptation.

Existing treatment planning methodologies demonstrate several critical limitations that affect treatment predictability and efficiency. First, they often operate in isolated domains, with orthodontic and surgical planning conducted separately rather than as an integrated process^18^. Second, current approaches tend to follow linear decision sequences that inadequately address the multifactorial nature of treatment response. Third, they typically lack systematic mechanisms for incorporating previous treatment outcomes as feedback for refining future treatment decisions. These limitations collectively contribute to suboptimal treatment outcomes, extended treatment durations, and occasional need for revision procedures.

The aforementioned challenges underscore the necessity for advanced computational approaches that can process multidimensional clinical data, recognize complex patterns in treatment responses, and generate personalized treatment strategies. Artificial intelligence technologies, particularly multi-task reinforcement learning and explainable AI models, offer promising frameworks for addressing these limitations by providing data-driven, adaptive, and transparent decision support tools for orthodontic-orthognathic treatment planning.

### Application of multi-task reinforcement learning in healthcare

Multi-task reinforcement learning (MTRL) extends traditional reinforcement learning by enabling agents to simultaneously learn and optimize multiple related tasks, making it particularly suitable for complex healthcare decision-making scenarios^19^. The fundamental mathematical framework of MTRL builds upon the Markov Decision Process (MDP), defined as a tuple $\:\left(S,A,P,R,\gamma\:\right)$, where $\:S$ represents the state space, $\:A$ the action space, $\:P$ the transition probability function, $\:R$ the reward function, and $\:\gamma\:$ the discount factor determining the importance of future rewards^20^. In the multi-task setting, this formulation expands to incorporate multiple reward functions $\:R=\{{R}_{1},{R}_{2},...,{R}_{n}\}$ corresponding to different clinical objectives.

The central goal of MTRL is to find an optimal policy $\:{\pi\:}^{\text{*}}$ that maximizes the expected cumulative reward across all tasks:

$$\:{\pi\:}^{\text{*}}=\text{a}\text{r}\text{g}\underset{\pi\:}{\text{m}\text{a}\text{x}}{\mathbb{E}}_{\tau\:}\left[\sum\:_{t=0}^{T}{\gamma\:}^{t}\sum\:_{i=1}^{n}{w}_{i}{R}_{i}\left({s}_{t},{a}_{t}\right)\right]$$

where $\:\tau\:$ represents trajectories of states and actions, $\:T$ is the time horizon, $\:{w}_{i}$ are importance weights for each task, and $\:{R}_{i}\left({s}_{t},{a}_{t}\right)$ is the reward for task $\:i$ at time step $\:t$^21^. This framework allows for the balancing of competing clinical objectives, such as treatment efficacy, patient comfort, and treatment duration.

In healthcare applications, MTRL algorithms often employ deep neural networks to approximate the Q-function, which represents the expected future reward of taking action $\:a$ in state $\:s$:

$$\:{Q}_{\theta\:}\left(s,a\right)=\mathbb{E}\left[\sum\:_{t=0}^{T}{\gamma\:}^{t}R\left({s}_{t},{a}_{t}\right)|{s}_{0}=s,{a}_{0}=a\right]$$

where $\:\theta\:$ represents the parameters of the neural network^22^. For multi-task scenarios, this extends to a vector of Q-functions:

$$\:{\mathbf{Q}}_{\theta\:}\left(s,a\right)=\left[{Q}_{\theta\:}^{1}\left(s,a\right),{Q}_{\theta\:}^{2}\left(s,a\right),...,{Q}_{\theta\:}^{n}\left(s,a\right)\right]$$

Policy gradient methods in MTRL optimize the policy directly by updating the policy parameters $\:\varphi\:$ according to:

$$\:{\nabla\:}_{\varphi\:}J\left(\varphi\:\right)={\mathbb{E}}_{{\pi\:}_{\varphi\:}}\left[{\nabla\:}_{\varphi\:}\text{l}\text{o}\text{g}{\pi\:}_{\varphi\:}\left(a|s\right)\sum\:_{i=1}^{n}{w}_{i}{Q}^{i}\left(s,a\right)\right]$$

This approach facilitates learning complex decision strategies that consider multiple treatment outcomes simultaneously^23^.

The application of MTRL in medical decision support systems has demonstrated significant advantages over traditional single-objective approaches, particularly in chronic disease management and treatment planning^24^. The multi-objective optimization capability enables clinicians to navigate complex trade-offs between immediate interventions and long-term outcomes, a critical consideration in orthodontic-orthognathic treatment where decisions on tooth movement, skeletal correction, and soft tissue adaptation are highly interdependent.

For orthodontic-orthognathic treatment specifically, the MTRL framework can be formulated to optimize a weighted combination of objectives:

$$\:J\left(\pi\:\right)={w}_{1}{J}_{functional}\left(\pi\:\right)+{w}_{2}{J}_{aesthetic}\left(\pi\:\right)+{w}_{3}{J}_{stability}\left(\pi\:\right)+{w}_{4}{J}_{duration}\left(\pi\:\right)$$

where each $\:{J}_{i}\left(\pi\:\right)$ represents a distinct clinical objective^25^. This formulation allows for personalization by adjusting weights based on patient-specific priorities and clinical constraints.

The transferability of learned policies between related tasks represents another significant advantage of MTRL in healthcare applications. Through shared representations, defined as:

$$\:\varphi\:\left(s\right)=\left[{h}_{\text{shared}}\left(s\right),{h}_{\text{task-specific}}\left(s\right)\right]$$

where $\:{h}_{\text{shared}}$ and $\:{h}_{\text{task-specific}}$ are shared and task-specific feature extractors respectively, MTRL can leverage commonalities across patient cases while adapting to individual variations^26^. This capability is particularly valuable in orthodontic-orthognathic treatment, where certain biomechanical principles remain consistent despite high patient variability.

### Role of explainable artificial intelligence in clinical decision support

Explainable Artificial Intelligence (XAI) has emerged as a critical component in healthcare artificial intelligence systems, addressing the fundamental challenge of algorithmic transparency in high-stakes medical decision-making^27^. While complex deep learning models have demonstrated remarkable predictive performance in numerous clinical applications, their inherent opacity has created significant barriers to clinical adoption and regulatory approval^28^. The “black-box” nature of many advanced AI algorithms contradicts the medical principle of informed decision-making, where clinicians must understand and communicate the rationale behind their treatment recommendations.

XAI methodologies can be broadly categorized into intrinsic and post-hoc approaches. Intrinsic methods incorporate transparency directly into model architecture through techniques such as attention mechanisms, sparse linear models, and rule-based systems that maintain interpretability by design^29^. Post-hoc methods, conversely, apply interpretation techniques to already trained models using approaches like Local Interpretable Model-agnostic Explanations (LIME), SHapley Additive exPlanations (SHAP), and feature importance visualizations^30^. These complementary approaches facilitate different levels of explanation granularity, from global model behavior understanding to local interpretation of individual predictions.

The evaluation of explainability in medical AI systems extends beyond technical metrics to encompass human-centered measures of comprehensibility and clinical utility^31^. Effective explanations must balance accuracy, fidelity to the underlying model, consistency across similar cases, and comprehensibility to the clinical end-user. This multifaceted evaluation approach recognizes that explanations serve diverse purposes in clinical workflows, including decision validation, clinical education, and patient communication about AI-assisted decisions.

Regulatory frameworks for medical AI increasingly emphasize explainability requirements, with the European Union’s General Data Protection Regulation (GDPR) establishing a “right to explanation” and the FDA developing guidance for AI/ML-based Software as a Medical Device (SaMD) that incorporates transparency considerations^32^. These regulatory trends align with emerging ethical frameworks that prioritize algorithmic transparency as a mechanism for preserving clinician autonomy and enabling informed patient consent in AI-assisted healthcare^33^.

In orthodontic-orthognathic treatment contexts, explainability assumes particular importance due to the long-term consequences of treatment decisions and the multidisciplinary nature of care delivery^34^. By providing transparent reasoning for treatment recommendations, XAI systems can facilitate communication between orthodontists, maxillofacial surgeons, and patients, enhancing collaborative decision-making while preserving the human expertise integral to treatment success.

The integration of XAI with reinforcement learning presents unique challenges stemming from the sequential nature of decision-making and the complexity of learned policies^35^. Approaches to explainable reinforcement learning include action influence models that quantify how specific states influence policy decisions, counterfactual explanations that demonstrate alternative treatment trajectories, and attention-based mechanisms that highlight salient features driving clinical recommendations^36^. These techniques can transform complex treatment policies into clinically interpretable guidance, bridging the gap between algorithmic optimization and evidence-based clinical practice.

## Methodology and system architecture

### Treatment planning framework based on Multi-Task reinforcement learning

The proposed framework conceptualizes orthodontic-orthognathic treatment planning as a sequential decision-making process, where clinical interventions are selected iteratively to optimize multiple treatment objectives simultaneously^37^. We formulate this clinical decision problem within a multi-task reinforcement learning (MTRL) paradigm using a Markov Decision Process (MDP) adapted for the specific requirements of dentofacial deformity correction. This approach enables the system to learn optimal treatment strategies directly from clinical data while balancing competing objectives such as functional improvement, aesthetic outcomes, treatment duration, and post-treatment stability.

The state space $\:S$ comprises comprehensive patient-specific features that characterize dentofacial morphology, tissue biomechanics, and treatment progress indicators^38^. Each state $\:{s}_{t}\in\:S$ at time step $\:t$ is represented as a high-dimensional vector:

$$\:{s}_{t}=\left[{s}_{t}^{skeletal},{s}_{t}^{dental},{s}_{t}^{soft\_tissue},{s}_{t}^{functional},{s}_{t}^{treatment\_status}\right]$$

where each component captures distinct but interrelated aspects of the patient’s condition. The granularity of this state representation enables precise modeling of individual variations in anatomical structure and treatment response.

The action space $\:A$ encompasses the complete spectrum of clinical interventions available in orthodontic-orthognathic treatment, including orthodontic mechanics, surgical procedures, and temporal sequencing decisions^39^. For a given state $\:{s}_{t}$, the agent selects an action $\:{a}_{t}\in\:A$ according to policy $\:\pi\:\left({a}_{t}|{s}_{t}\right)$. Table 1 defines the specific state and action spaces for key treatment tasks in the combined therapy.

**Table 1:** Definition of state space and action space.

Treatment Task Type	State Space Representation	Action Space Definition
Jaw Position Adjustment	3D skeletal landmarks (68 points), cephalometric measurements (SNA, SNB, ANB), occlusal relationship (Class I/II/III)	Surgical movement vectors (± 15 mm) in sagittal, vertical (± 10 mm), and transverse dimensions (± 8 mm)
Dental Alignment Optimization	Tooth positions (x, y,z coordinates), angulations (tip/torque), rotations; arch form parameters (width, length)	Force systems (50–300 g magnitude, 3D direction vectors), appliance selection (brackets, wires, elastics), activation timing
Soft Tissue Management	Tissue thickness maps (64 measurement points), functional parameters (lip competence, nasal breathing), aesthetic indices (facial harmony score 0–10)	Surgical technique selection (V-Y advancement, reduction), soft tissue handling protocols, timing of interventions (simultaneous/staged)
Treatment Sequencing	Current treatment phase (1–4 scale), stability metrics (relapse risk 0–1), healing indicators (bone density, tissue adaptation)	Phase transition decisions (proceed/hold/modify), treatment cadence adjustments (4–8 week intervals), intervention scheduling (priority matrix)

Values in parentheses indicate measurement ranges or scales used in system implementation.

The multi-objective reward function $\:R\left({s}_{t},{a}_{t},{s}_{t+1}\right)$ quantifies the clinical value of transitioning from state $\:{s}_{t}$ to $\:{s}_{t+1}$ after executing action $\:{a}_{t}$^40^. This function integrates multiple treatment objectives through a weighted combination:

$$\:R\left({s}_{t},{a}_{t},{s}_{t+1}\right)=\sum\:_{i=1}^{n}{w}_{i}\cdot\:{r}_{i}\left({s}_{t},{a}_{t},{s}_{t+1}\right)$$

where $\:{r}_{i}$ represents individual reward components corresponding to specific clinical goals, and $\:{w}_{i}$ are importance weights determined through clinical expert consensus and patient preference elicitation^41^. The specific reward components include:

$$\:{r}_{functional}=f\left(\text{occlusal\:function},\text{TMJ\:health},\text{respiratory\:parameters}\right)$$

$$\:{r}_{aesthetic}=f\left(\text{facial\:harmony},\text{smile\:aesthetics},\text{profile\:balance}\right)$$

$$\:{r}_{efficiency}=f\left(\text{treatment\:duration},\text{appointment\:frequency},\text{resource\:utilization}\right)$$

$$\:{r}_{stability}=f\left(\text{relapse\:potential},\text{tissue\:adaptation},\text{growth\:consideration}\right)$$

The optimization of this framework employs a modified Soft Actor-Critic (SAC) algorithm, chosen for its sample efficiency and stability in continuous action spaces^42^. The policy network $\:{\pi\:}_{\varphi\:}\left(a|s\right)$ and critic networks $\:{Q}_{{\theta\:}_{1}}\left(s,a\right)$ and $\:{Q}_{{\theta\:}_{2}}\left(s,a\right)$ are parameterized by deep neural networks with architecture specifically designed to capture the hierarchical relationships between skeletal, dental, and soft tissue components.

Training of this MTRL framework utilizes a combination of retrospective clinical data from successfully treated cases and simulated treatment scenarios generated through biomechanical modeling^43^. The learned policy iteratively improves through experience, incorporating both positive outcomes and treatment complications as valuable learning signals. This approach enables the system to develop robust treatment strategies that adapt to individual patient characteristics while maintaining alignment with established clinical principles.

### Explainable AI component design and integration

The clinical decision support platform incorporates multiple complementary explainable AI (XAI) components that render the underlying reinforcement learning model transparent and interpretable to orthodontic and maxillofacial specialists^44^. These explainability layers function as cognitive bridges between complex computational models and clinical reasoning patterns, enabling practitioners to understand, validate, and when necessary, override AI-generated treatment recommendations. Table 2 presents a comparison of the explainable AI methods integrated into the system architecture.

**Table 2:** Comparison of explainable AI Methods.

Explainable Method	Method Principle	Applicable Scenarios	Application in Orthodontic-Orthognathic Treatment
Feature Attribution Analysis	Quantifies contribution of input features to model predictions using gradient-based or perturbation methods	Understanding feature influence in complex models with high-dimensional inputs	Highlighting critical cephalometric parameters and morphological features driving specific treatment recommendations
Decision Tree Approximation	Extracts simplified decision rules from complex models by training interpretable surrogates	Policy distillation and global model explanation	Translating learned treatment policies into clinical guidelines with explicit condition-action rules
Attention Visualization	Maps model attention to anatomical regions or clinical parameters during decision-making	Spatial data interpretation and feature localization	Visualizing focus areas on 3D craniofacial models during treatment planning and surgical simulation
Counterfactual Explanation	Generates alternative scenarios by minimal feature modifications to change model output	What-if analysis and treatment alternative exploration	Demonstrating how specific modifications to treatment parameters would affect predicted outcomes
Treatment Trajectory Explanation	Decomposes sequential decisions into clinically meaningful phases with outcome justifications	Temporal process interpretation and long-term planning	Explaining the rationale behind treatment sequencing decisions and phase transitions

The feature attribution module implements integrated gradients and SHAP (SHapley Additive exPlanations) algorithms using Python SHAP v0.41.0 and Captum v0.6.0 libraries to identify the relative importance of craniofacial features in treatment decisions^45^. LIME (Local Interpretable Model-agnostic Explanations) v0.2.0.1 provides additional local explanations for individual treatment recommendations. This component transforms complex network activations into clinically interpretable visualizations that highlight influential anatomical structures and occlusal parameters on 3D patient models. The implementation includes domain-specific adaptations to account for the hierarchical relationships between skeletal, dental, and soft tissue components in orthodontic-orthognathic treatment.

Decision path visualization provides clinicians with transparent access to the model’s reasoning process through graphical representations of the decision sequence^46^. This component maps the reinforcement learning agent’s state-action trajectory onto established clinical workflows, enabling practitioners to recognize familiar treatment patterns while identifying novel approaches suggested by the AI system. The visualization adapts its complexity based on user expertise, offering detailed technical explanations for specialists and simplified representations for interdisciplinary communication.

Counterfactual explanation generation addresses the critical “what-if” questions central to clinical decision-making by simulating alternative treatment scenarios^47^. This component identifies minimal modifications to treatment parameters that would result in significantly different outcomes, allowing clinicians to explore the boundaries of treatment possibilities and understand outcome sensitivities. The system generates these explanations through constrained optimization within clinically valid parameter ranges, ensuring that all alternatives remain biologically feasible.

The integrated explanatory system employs a multi-level abstraction architecture that translates technical model outputs into contextually appropriate clinical narratives^48^. At the highest level, natural language explanations articulate treatment rationales using specialty-specific terminology aligned with orthodontic and surgical conventions. These explanations are dynamically generated through a template-based approach that maps model states and actions to clinical concepts while maintaining consistency with visual explanations.

The bidirectional integration between the explainable components and the reinforcement learning core enables not only post-hoc interpretation but also interactive refinement of treatment plans. Clinician feedback on explanations is systematically incorporated into model training through a hybrid human-AI learning loop, progressively aligning the system’s decision-making with expert clinical judgment while preserving its ability to identify novel treatment approaches based on comprehensive data analysis.

### Overall architecture of clinical decision support platform

The clinical decision support platform integrates multiple computational components within a coherent system architecture designed to facilitate seamless workflow integration in orthodontic and maxillofacial surgery practices^49^. The platform adopts a modular microservices architecture that enables independent development, testing, and updating of individual components while maintaining system-wide interoperability. Table 3 summarizes the principal system components and their respective functions.

**Table 3:** System components and functions.

System Component	Technical Implementation	Main Functional Description
Data Integration Engine	HL7 FHIR-compliant ETL pipeline with vendor-specific adapters	Extracts and normalizes heterogeneous clinical data from CBCT, intraoral scanners, electronic health records, and treatment planning software
Patient Digital Twin	Graph-based representation with hierarchical mesh models and biomechanical simulation capabilities	Creates comprehensive digital patient representation for treatment simulation and outcome prediction with real-time updatability
MTRL Treatment Optimization Core	Distributed computing infrastructure with GPU acceleration and model parallelization	Executes reinforcement learning algorithms to generate and refine personalized treatment plans based on patient-specific data
XAI Interpretation Layer	Modular explanation generators with domain-specific visualization engines	Transforms complex model decisions into clinically interpretable explanations through multiple complementary methods
Collaborative Decision Interface	Progressive web application with responsive design and role-based access control	Provides customized interfaces for different clinical roles with interactive visualization and modification capabilities
Security and Compliance Framework	Blockchain-based audit logging and federated learning architecture	Ensures data privacy, regulatory compliance, and maintains auditability of all system operations

The platform implements a bidirectional data flow that begins with the secure acquisition of patient diagnostic records through the Data Integration Engine, which employs HL7 FHIR (Fast Healthcare Interoperability Resources) standards to establish interoperability with existing hospital information systems and imaging workstations^50^. System performance analysis showed average processing time of 8.4 ± 2.1 min per case on standard clinical workstations (Intel i7-10700 K, 32GB RAM, NVIDIA RTX 3080), with cloud deployment options available for institutions with limited computational resources. The platform supports standard medical imaging formats (DICOM, STL, PLY) and integrates with common orthodontic software including Dolphin Imaging, 3Shape, and Planmeca systems. This standardized approach minimizes implementation barriers while maintaining compliance with international healthcare data exchange protocols.

The user interface employs a clinician-centered design philosophy that adapts to different user roles and expertise levels through contextually sensitive information presentation^51^. For orthodontists, the interface emphasizes dental alignment sequencing and biomechanical considerations, while maxillofacial surgeons receive focused visualizations of skeletal relationships and surgical simulation outcomes. This role-adaptive approach extends to the explanation generation, which adjusts terminology and detail levels based on user expertise and specific information needs.

Data security and privacy protection are implemented through a multi-layered approach that combines traditional encryption methods with advanced federated learning techniques that enable model training without centralized storage of sensitive patient information^52^. The platform maintains comprehensive audit logs of all system interactions using blockchain technology, ensuring non-repudiation and traceability of clinical decisions while supporting regulatory compliance with regional healthcare data protection frameworks including HIPAA, GDPR, and their international equivalents.

The collaborative decision-making workflow orchestrates interactions between clinicians and AI components through a structured yet flexible process that preserves clinical autonomy while leveraging computational capabilities. Initially, the system generates multiple treatment scenarios based on patient-specific data, presenting these alternatives with corresponding explanations and predicted outcomes. Clinicians can interactively modify these recommendations through direct manipulation of 3D models or parameter adjustments, with the system providing real-time feedback on the implications of proposed changes. This iterative refinement process culminates in a finalized treatment plan that represents a true collaboration between human expertise and artificial intelligence, documented with comprehensive justifications that support clinical implementation and patient communication.

## Experimental validation and result analysis

### Experimental setup and dataset description

The experimental validation utilized a comprehensive retrospective dataset comprising 347 orthodontic-orthognathic patients treated between 2015 and 2023 at three university-affiliated craniofacial centers^53^.

Patient inclusion criteria required complete pre-treatment records (lateral and frontal cephalometric radiographs, CBCT scans, intraoral and facial photographs, and digital dental models), documented treatment plans, sequential progress records, and minimum two-year post-treatment follow-up data. Patients were predominantly post-pubertal adults (mean age 22.7 ± 4.3 years, range 18–35 years) with completed skeletal growth as confirmed by cervical vertebral maturation (CVM) stage V-VI. Growing patients were excluded from this initial validation to focus on treatment planning without growth prediction variables, though future development will incorporate growth forecasting algorithms for adolescent applications.

Exclusion criteria encompassed patients with craniofacial syndromes, cleft lip/palate, systemic conditions affecting bone metabolism, incomplete treatment documentation, or discontinued treatment protocols. Table 4 summarizes the demographic and clinical characteristics of the included patient population.

**Table 4:** Patient demographics and clinical features.

Feature Category	Mean/Frequency	Standard Deviation/Percentage	Clinical Significance
Age at Treatment Initiation	22.7 years	± 4.3 years	Skeletal maturity status influencing treatment approach and stability
Gender Distribution	Female: 198, Male: 149	57.1% / 42.9%	Sex-based dimorphism in craniofacial morphology and treatment response
Dentofacial Deformity Classification	Class II: 142, Class III: 187, Asymmetry: 132, Vertical Discrepancy: 95	40.9% / 53.9% / 38.0% / 27.4%	Primary skeletal discrepancy pattern determining surgical approach and sequencing
Pre-treatment Cephalometric Values	ANB: -2.8° (Class III), 5.9° (Class II)	± 2.1° / ±1.8°	Sagittal jaw relationship severity affecting complexity of correction required
Treatment Duration	26.3 months	± 6.7 months	Temporal efficiency metric for treatment optimization

Data preprocessing followed a standardized pipeline that included anatomical landmark identification on CBCT volumes, registration of multimodal imaging data to a common coordinate system, and extraction of 137 craniofacial measurements^54^. The landmark detection employed a modified U-Net architecture with ResNet-50 backbone, trained on 2,847 manually annotated CBCT scans with 68 anatomical landmarks per case. The 137 measurements comprised: 45 skeletal parameters (including SNA, SNB, ANB angles and facial heights), 52 dental measurements (tooth positions, angulations, and arch dimensions), and 40 soft tissue metrics (profile angles and tissue thickness measurements). A semi-automated approach utilizing this deep learning-based landmark detection with expert verification ensured measurement accuracy, with intra-examiner and inter-examiner reliability coefficients exceeding 0.92 for all primary variables and landmark detection accuracy of 94.3% within 2 mm clinical tolerance. Longitudinal data were temporally aligned relative to treatment milestones (pre-surgical orthodontics initiation, surgery date, post-surgical orthodontics completion) to facilitate sequential modeling.

The dataset was partitioned chronologically, with 70% allocated for model training (*n* = 243), 15% for validation during development (*n* = 52), and 15% for final performance testing (*n* = 52). This temporal splitting strategy preserves the clinical realism of the evaluation by ensuring that predictions are made only using historically available data, mirroring real-world implementation conditions^55^. Treatment outcomes were categorized according to established clinical criteria including occlusal function metrics, cephalometric parameter normalization, facial aesthetic improvements, and post-treatment stability indices.

Model training employed a five-fold cross-validation protocol within the training dataset, with hyperparameter optimization guided by performance on the validation subset. Early stopping criteria based on validation performance prevented overfitting, while automated data augmentation techniques including small random perturbations of landmark positions enhanced model robustness to measurement variability. Evaluation metrics were selected to address both technical performance (prediction accuracy, policy consistency) and clinical relevance (treatment duration optimization, outcome quality assessment by blinded experts)^56^.

All data collection and usage procedures were approved by the institutional ethics committees of participating centers (approval numbers: CREEC-2021-078, ORTH-IRB-2020-134, MFOS-EC-2022-056), with written informed consent obtained from all patients for retrospective data utilization in research. Data anonymization protocols removed all personally identifiable information prior to inclusion in the research database, and all analyses were conducted in compliance with relevant data protection regulations.

### Performance evaluation and comparative analysis

The proposed multi-task reinforcement learning-based decision support platform was evaluated against traditional treatment planning approaches and existing single-objective AI methods across multiple performance dimensions^57^. The baseline AI method employed a modified ResNet-101 architecture adapted for orthodontic treatment prediction, trained using standard supervised learning with single-objective optimization focusing solely on treatment duration minimization. This baseline was implemented with Adam optimizer (learning rate = 0.001), batch size of 32, and 200 training epochs, following similar approaches reported in orthodontic AI literature^58,59^.

Traditional treatment planning followed established clinical protocols including ABO (American Board of Orthodontics) guidelines and standardized surgical planning procedures. Conventional plans were developed by experienced orthodontists (> 10 years experience) and oral-maxillofacial surgeons using lateral cephalometric analysis, clinical examination, and 3D imaging interpretation. The planning process followed a sequential approach: initial orthodontic alignment, pre-surgical preparation, surgical simulation using commercial software (Dolphin Imaging v11.9), and post-surgical finishing protocols. Inter-examiner agreement for traditional planning showed kappa coefficients of 0.78–0.85 across different treatment decisions. Comprehensive assessment metrics were defined to capture both computational efficiency and clinical relevance, with statistical significance determined using paired t-tests with Bonferroni correction for multiple comparisons.

Table 5 summarizes the comparative performance results across key evaluation criteria.

**Table 5:** Performance comparison with baseline methods.

Evaluation Metric	Proposed Method	Traditional Method	Other AI Method	Statistical Significance
Treatment Plan Quality Score (0-100)	87.3 ± 6.2	72.8 ± 9.5	79.4 ± 8.1	*p* < 0.001
Decision Time (minutes)	12.4 ± 3.1	47.6 ± 12.5	28.3 ± 8.7	*p* < 0.001
Prediction Accuracy (%)	92.7 ± 3.8	N/A	84.1 ± 5.3	*p* < 0.01
Expert Agreement Rate (%)	86.5 ± 7.2	77.3 ± 11.4	80.6 ± 9.5	*p* < 0.05

The quantitative evaluation employed a composite Treatment Plan Quality Score (TPQS) calculated as:

$$\:\text{TPQS}=\sum\:_{i=1}^{n}{w}_{i}\cdot\:{Q}_{i}$$

where $\:{Q}_{i}$ represents individual quality components with specific measurement protocols: occlusal function (0–25 points, based on ABO Objective Grading System criteria including overjet, overbite, and posterior contacts), aesthetic improvement (0–25 points, using validated facial attractiveness scales and smile analysis), biomechanical efficiency (0–25 points, quantified by force system optimization and treatment duration), and stability prediction (0–25 points, assessed through finite element analysis of relapse potential). Component weights were: $\:{w}_{1}=0.30$ (function), $\:{w}_{2}=0.25$ (aesthetics), $\:{w}_{3}=0.25$ (efficiency), $\:{w}_{4}=0.20$ (stability), determined through an Analytic Hierarchy Process involving 15 senior clinicians^60^.

The proposed system demonstrated a 19.9% improvement in TPQS compared to traditional planning methods and a 9.9% improvement over single-objective AI approaches.

Prediction accuracy was assessed using a temporally stratified cross-validation approach, measuring the system’s ability to forecast treatment outcomes at multiple intervals:

$$\:\text{Accuracy}\left(t\right)=1-\frac{1}{m}\sum\:_{j=1}^{m}\frac{\left|{\widehat{y}}_{j}\left(t\right)-{y}_{j}\left(t\right)\right|}{{y}_{j}\left(t\right)}$$

where $\:{\widehat{y}}_{j}\left(t\right)$ represents the predicted value for outcome measure $\:j$ at time point $\:t$, and $\:{y}_{j}\left(t\right)$ represents the corresponding actual clinical value^61^. The multi-task approach consistently outperformed single-objective methods, with particular advantages in predicting long-term stability metrics.

Treatment efficiency gains were quantified using a normalized efficiency index:

$$\:\text{Efficiency}=\frac{{Q}_{outcome}}{\varDelta\:{T}_{treatment}\cdot\:{C}_{resources}}$$

where $\:{Q}_{outcome}$ represents outcome quality, $\:\varDelta\:{T}_{treatment}$ is treatment duration, and $\:{C}_{resources}$ represents resource utilization^62^. The proposed system achieved an average reduction of 3.7 months in total treatment duration while maintaining or improving outcome quality.

The clinical confidence score, measuring clinician trust in system recommendations, was calculated as:

$$\:\text{Confidence}=\alpha\:\cdot\:\text{Explainability}+\beta\:\cdot\:\text{EvidenceStrength}+\gamma\:\cdot\:\text{ConsensusAlignment}$$

with standardized weights $\:\alpha\:$, $\:\beta\:$, and $\:\gamma\:$ determined through clinician surveys. Explainability features contributed significantly to higher confidence scores compared to black-box alternatives^63^.

Performance stratification by case complexity revealed differential advantages across deformity types. For Class III skeletal patterns, the system demonstrated the highest performance improvement (24.3% over traditional methods), attributed to the complex interplay of sagittal, vertical, and transverse correction requirements in these cases. Asymmetry cases showed moderate improvement (18.7%), while Class II cases showed the smallest though still significant differential (11.5%). This pattern correlates with case difficulty rankings in orthodontic literature and suggests that AI assistance provides greater value in more complex clinical scenarios.

The system’s real-time biomechanical simulation capabilities enabled accurate prediction of tissue responses according to:

$$\:\text{Response}\left(t\right)=f\left(\text{initial\:state},\text{intervention\:parameters},\text{tissue\:properties},t\right)$$

This predictive modeling demonstrated particular advantages in complex cases requiring simultaneous optimization of multiple treatment objectives, where conventional planning approaches often rely on oversimplified heuristics and limited consideration of objective tradeoffs.

### Clinical case studies and expert feedback

To evaluate the clinical utility of the developed platform, we conducted an in-depth analysis of eight representative cases exhibiting diverse dentofacial deformity patterns^64^. Case selection criteria prioritized diagnostic complexity, treatment planning challenges, and outcome variability to provide comprehensive system testing across the clinical spectrum. For each case, treatment plans generated by the AI platform were compared with historical plans created through conventional methods by the treating clinicians.

A particularly illustrative case involved a 23-year-old female patient presenting with skeletal Class III malocclusion complicated by facial asymmetry and vertical maxillary deficiency. The AI-assisted planning process integrated cephalometric analysis, 3D facial scanning, and dental model data to simulate 18 potential treatment scenarios with varying surgical approaches and orthodontic mechanics. The system-recommended approach involved differential maxillary impaction with mandibular rotation and asymmetric setback, accompanied by specific torque prescriptions for anterior teeth to optimize dental compensation. The explainable AI component provided visualization of predicted soft tissue changes and biomechanical stress distributions, with counterfactual explanations demonstrating outcome variations under alternative approaches.

The platform’s recommendation database was analyzed by an expert panel comprising six orthodontists, four oral-maxillofacial surgeons, and two prosthodontists who evaluated system performance across multiple clinical dimensions^65^. The evaluation protocol included both quantitative scoring and qualitative feedback collection through structured interviews and usability testing sessions. Table 6 summarizes the expert evaluation results across the primary assessment dimensions.

**Table 6:** Expert evaluation results.

Evaluation Dimension	Orthodontists (*n* = 6)	Oral-Maxillofacial Surgeons (*n* = 4)	Prosthodontists (*n* = 2)
Treatment Plan Rationality	8.7/10 - “Comprehensive biomechanical considerations with innovative sequencing solutions”	8.3/10 - “Surgical planning demonstrates awareness of anatomical constraints and technical feasibility”	7.9/10 - “Good occlusal planning but occasional underemphasis on prosthetic considerations”
Decision Efficiency	9.2/10 - “Significant reduction in planning time for complex cases”	8.8/10 - “Streamlined surgical planning process with effective visualization tools”	8.5/10 - “Efficient integration of restoration requirements into overall treatment”
Explainability	8.5/10 - “Clear visualization of force systems and expected tooth movement”	7.9/10 - “Good explanation of skeletal movements, could improve soft tissue prediction clarity”	8.2/10 - “Effective communication of occlusal relationships and functional considerations”
Clinical Value	8.8/10 - “High potential for training and complex case management”	8.6/10 - “Valuable for surgical planning and patient communication”	8.1/10 - “Useful for interdisciplinary communication and treatment coordination”

Expert feedback highlighted several key advantages of the AI-assisted approach, particularly in complex cases requiring careful balance between multiple treatment objectives^66^. Orthodontists emphasized the system’s ability to identify efficient mechanical solutions and optimize treatment sequencing, while surgeons valued the precise surgical simulation capabilities and biomechanical validity of recommended approaches. The explainability features received positive evaluation across all specialist groups, with particular appreciation for the ability to visualize treatment alternatives and their predicted outcomes during patient consultations.

Areas identified for improvement included the need for more comprehensive consideration of growth modification in adolescent patients, enhanced integration of temporomandibular joint dynamics in surgical planning, and more detailed articulation of prosthetic rehabilitation requirements in cases involving missing teeth or extensive restorations. These suggestions have been incorporated into the ongoing system refinement process to enhance clinical applicability across diverse patient populations.

## Conclusion and future prospects

This research presents a novel computational framework for orthodontic-orthognathic treatment planning that integrates multi-task reinforcement learning with explainable artificial intelligence to address the complex challenges of personalized treatment design. The developed platform demonstrates significant advancements in several key areas: (1) formulation of orthodontic-orthognathic treatment as a sequential decision-making problem amenable to reinforcement learning optimization; (2) implementation of a multi-objective reward structure that balances functional, aesthetic, efficiency, and stability considerations; (3) integration of complementary explainability methods that render complex AI recommendations interpretable within clinical contexts; and (4) creation of a collaborative decision support interface that enhances rather than replaces expert clinical judgment.

The multi-task reinforcement learning approach has demonstrated particular value in resolving the competing objectives inherent in orthodontic-orthognathic treatment planning. By simultaneously optimizing multiple treatment parameters across different time horizons, the system can identify treatment strategies that achieve superior outcomes compared to traditional sequential planning methods. The performance improvements were most pronounced in complex cases featuring multiple dentofacial deformities, where the interdependencies between treatment decisions are most challenging to navigate using conventional approaches. The system’s ability to leverage longitudinal treatment data enables continual refinement of predictive models and treatment policies, creating a virtuous cycle of improvement as additional clinical cases are incorporated.

The explainable AI components have proven essential for clinical acceptance and integration, transforming the platform from a theoretical advancement to a practical clinical tool. By providing transparent reasoning for treatment recommendations, the system supports informed clinical decision-making while facilitating effective communication between specialists and with patients. The complementary explanation modalities address different aspects of clinical comprehension, from biomechanical understanding to treatment sequencing rationales, creating a comprehensive explanatory framework that builds clinician trust and confidence^67^.

Despite the promising results, several limitations warrant acknowledgment and represent opportunities for future research. First, the current validation is limited to retrospective data analysis, requiring prospective clinical trials to establish real-world effectiveness and safety. A multi-center prospective study involving 200 patients is planned for 2025–2026 to validate clinical outcomes and workflow integration. Second, the current system’s predictive models require further validation across more diverse patient populations, particularly regarding growth prediction in adolescent patients and long-term stability assessment beyond two years. Third, computational requirements may limit accessibility in resource-constrained clinical settings, though cloud-based deployment strategies are being developed to address this limitation. Second, the computational demands of real-time treatment simulation present challenges for widespread clinical deployment, necessitating optimization of algorithmic efficiency and resource utilization. Third, integration with existing clinical workflows requires careful attention to user experience design and interoperability with established medical record systems.

Future research directions should address these limitations through several avenues: (1) expansion of training datasets to improve generalization across diverse patient populations and treatment protocols; (2) development of more sophisticated biomechanical simulation models that incorporate tissue adaptation and growth modification; (3) implementation of federated learning approaches that enable model improvement while preserving patient privacy; and (4) conduct of prospective clinical trials comparing AI-assisted treatment planning with conventional approaches^68^. Additionally, exploration of transfer learning techniques may enable adaptation of the developed framework to adjacent domains in dental medicine, including complex prosthodontic rehabilitation and craniofacial reconstruction.

The integration of artificial intelligence into orthodontic-orthognathic treatment represents a paradigm shift in clinical practice, moving from experience-based to evidence-driven decision-making while preserving the irreplaceable value of clinical expertise. By augmenting clinician capabilities with computational intelligence and enhancing treatment predictability through personalization, this approach has the potential to significantly improve treatment outcomes, efficiency, and patient satisfaction in the management of dentofacial deformities.

## Data Availability

All data included in this study are available upon request by contact with the corresponding author.
